# Review on Sublethal Effects of Environmental Contaminants in Honey Bees (*Apis mellifera*), Knowledge Gaps and Future Perspectives

**DOI:** 10.3390/ijerph18041863

**Published:** 2021-02-14

**Authors:** Agata Di Noi, Silvia Casini, Tommaso Campani, Giampiero Cai, Ilaria Caliani

**Affiliations:** 1 Department of Life Sciences, University of Siena, via Mattioli, 4, 53100 Siena, Italy; agata.dinoi@student.unisi.it (A.D.N.); cai@unisi.it (G.C.); 2 Department of Physical, Earth and Environmental Sciences, University of Siena, via Mattioli, 4, 53100 Siena, Italy; campani@unisi.it (T.C.); caliani4@unisi.it (I.C.)

**Keywords:** honey bees, sublethal effects, plant protection products, bees decline, monitoring strategies, methodological approach

## Abstract

Honey bees and the pollination services they provide are fundamental for agriculture and biodiversity. Agrochemical products and other classes of contaminants, such as trace elements and polycyclic aromatic hydrocarbons, contribute to the general decline of bees’ populations. For this reason, effects, and particularly sublethal effects of contaminants need to be investigated. We conducted a review of the existing literature regarding the type of effects evaluated in *Apis mellifera*, collecting information about regions, methodological approaches, the type of contaminants, and honey bees’ life stages. Europe and North America are the regions in which *A. mellifera* biological responses were mostly studied and the most investigated compounds are insecticides. *A. mellifera* was studied more in the laboratory than in field conditions. Through the observation of the different responses examined, we found that there were several knowledge gaps that should be addressed, particularly within enzymatic and molecular responses, such as those regarding the immune system and genotoxicity. The importance of developing an integrated approach that combines responses at different levels, from molecular to organism and population, needs to be highlighted in order to evaluate the impact of anthropogenic contamination on this pollinator species.

## 1. Introduction

Honey bees (*Apis mellifera*) are essential organisms for the environment, in particular for their critical roles in the pollination of crops, flowers, and fruit trees [1,2,3]. It has been estimated that honey bees are responsible for providing a pollination service to 96% of animal-pollinated crops [4,5]. Bees are also indirectly responsible for the reproduction and maintenance of wild plant communities and biodiversity [6,7,8]. Their value to global food crops is estimated at €153 billion per year [9]. In addition, honey bees provide honey, pollen, wax, propolis, and royal jelly to humans [10]. Throughout the last decade, declines in bees and other pollinators have been observed globally [11,12,13]; important honey bee colony losses have been reported, particularly in North America and Western Europe [14,15,16]. It was beekeepers who alerted the scientific community of this vital colony mortality, since they monitor bee colonies worldwide and are immediately aware of any kind of changes to the bees’ colony [17]. This decline has led to concerns over there being a sustainable food supply and the health of natural ecosystems [18]. The causes of pollinator decline may be complex and subject to disagreement. However, the general weakening and death of bee colonies has been observed to be mainly caused by the combined effects of multiple stressors [3,19,20,21], such as biological factors [22,23], environmental factors [19,24,25], chemical and nutritional stressors [26,27], chemical and biological factors [28,29,30,31,32,33] and multiple chemicals [34,35,36]. In particular, this last kind of stressor is a matter of great concern since bees can be exposed to a wide range of chemical mixtures, including anthropogenic compounds, such as plant protection products (PPPs) or veterinary drugs, and those of natural origin, such as mycotoxins, flavonoids and plant toxins [20,37,38]. Although PPPs, such as insecticides, acaricides, herbicides, and fungicides, have many benefits for agriculture [39], there are also several potential risks associated with their use, such as pest resistance, resurgence, and secondary pest outbreaks, as well as wider environmental contamination and human health concerns [40,41,42]. Although insecticides are applied to target insect pests, their use in agriculture can affect non-target insects that provide beneficial services to agriculture. Among these beneficial insects, the focus was on social bees, with a particular interest in neonicotinoid insecticides and their lethal and sublethal effects at colony and population levels. Nonetheless, other PPPs used in modern agriculture, such as fungicides and herbicides, were demonstrated to affect honey bee’s health status [43,44,45,46].

The sublethal effects of PPPs and other anthropogenic contaminants in *Apis mellifera* need to be investigated. A wide range of studies investigated mortality and accumulation in honey bees, in order to obtain data related to contamination that may affect these organisms [33,47,48,49]. Moreover, studies concerning the general fitness of honey bees, which examined their behaviour, flight activity, and sensory ability, were conducted over the years to observe the macroscopic effects of contaminants [48,50,51,52]. To a lesser extent, enzymatic and molecular responses have also been studied, using genomic, metabolomic, and transcriptomic techniques and biomarkers [43,53,54,55,56], in order to increase understanding of the anthropogenic impact on these insects.

The current manuscript aims to provide a review of the available toxicological studies about the biological responses of honey bees to external stressors. In particular, we focused on where studies were carried out, we examined the contaminants involved, methodological approaches, honey bees’ life stages, and the different kind of responses considered in each paper, with the purpose to determine and identify knowledge gaps. This review could also provide indications regarding possible improvements in the monitoring approach, both in a scientific and regulatory way.

## 2. Materials and Methods

The search for scientific papers was conducted on ScienceDirect, Google Scholar, and One search database, using the following search terms to find relevant literature: “*Apis mellifera*”, “honey bees”, “biomarkers”, “ecotoxicology”, “toxicology”, “sublethal effects”, and “biochemical analysis”. To extend the collection of the relevant literature, the bibliographical references of each article were also examined. The selected articles were written in English and the full text version is available. Grey literature and non-accessible peer-reviewed articles were not included in our work, and this resulted in a primary dataset of 846 publications.

Papers considered for this review included investigations into toxicity effects, sublethal behavioural effects, impacts on bees at a genetic, molecular, or physiological level. Studies that reported only LC50 and LD50 were omitted from our analysis. The final dataset included a total of 106 research papers. For each paper, we extracted the following information: a complete bibliographical reference, a methodological approach, the investigated compounds, the life stage, and the studied responses. Where multiple categories of any variable were reported in the same paper, all were included in the final analyses. Methodological approaches were divided into three categories: “laboratory”, “semi-field” and “field”. “Laboratory” studies were defined as those carried out within the laboratory, with the exposure of honey bees to contaminants. “Semi-field” studies were defined as those that were conducted outdoors, but confined to bees, e.g., using exclusion cages. “Field” studies were defined as studies conducted outdoors with no restriction on the bees’ movements and the data were collected in the field.

The compounds studied in the papers were divided into insecticides, herbicides, fungicides, acaricides, trace elements, polycyclic aromatic hydrocarbons (PAHs), parasites, radioactivity, mixtures, and other compounds.

The following life stages were considered: “Brood”, “Pupae”, “Larvae”, “Adults” and “Queens”. If the life stages at which bees were exposed to pesticides differed from the life stage at which the effects were measured, then both were included in the final analyses.

Examining the existing literature, we described fifteen different “effect types” that were assessed, including morphology, apoptosis and necrosis, histopathology, cytotoxicity, consumption, foraging activity, and fitness, learning ability, other behaviours, physiological function and morphology, reproduction, sensory (gustatory or olfactory), flight activity, growth and development and, accumulation. Research studies were placed into multiple categories if they contained more than one effect type.

Moreover, we isolated more specific responses, mostly characterized by biomarkers and transcriptomic, metabolomic, proteomic approaches, in nine endpoints: detoxification, neurotoxicity, immunity, metabolism, oxidative stress, genotoxicity, primary stress response, carbohydrates assay, and protein amounts. Where studies included more than one option in any of the variables measured, it was included in analyses of both.

## 3. Results

### 3.1. Where Studies Took Place

**Figure 1:**
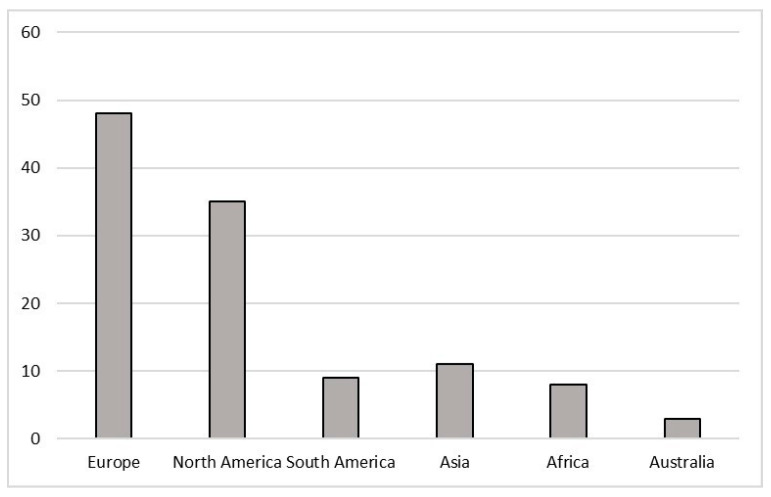
Number of studies, conducted on *Apis mellifera*, and divided by continent, that met the criteria for inclusion in this review.

Most studies examined for this review were carried out in Europe (48) and North America (35), followed by Asia (11) and South America (9), Africa (8) and Australia (3) (Figure 1).

### 3.2. Methodological Approaches

**Figure 2:**
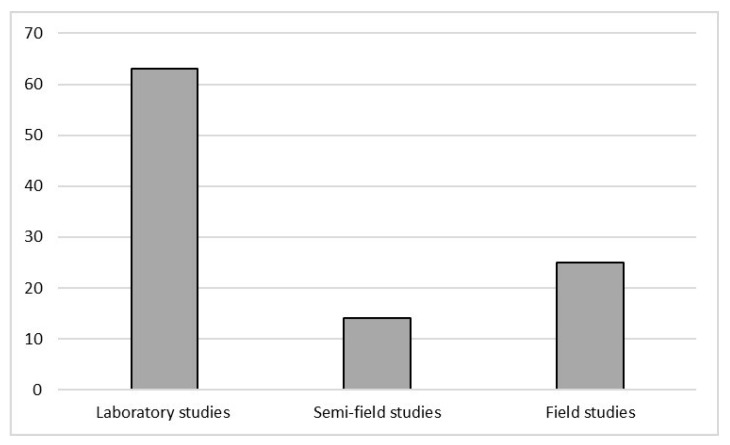
Number of studies, divided by a methodological approach, on *Apis mellifera*, that met the criteria for inclusion in this review.

As shown in Figure 2, most studies were carried out under laboratory conditions (63), with 14 studies carried out in semi-field conditions, and 25 at the full field scale.

### 3.3. Life Stages

**Figure 3:**
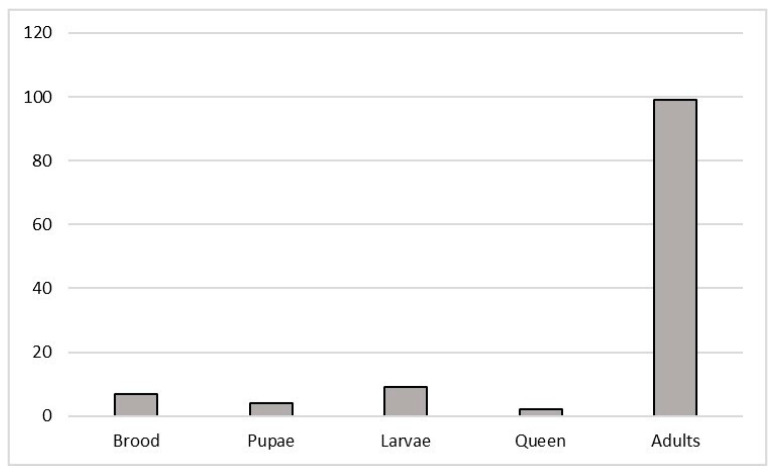
Number of studies, divided by life stages, on *Apis mellifera*, that met the criteria for inclusion in this review.

The bibliographical research highlighted that most of the studies, as shown in Figure 3, were conducted on adult bees (99), followed by larvae (9), brood (7), and pupae stage (4). Only 2 studies, that met the criteria of this work, were about queen bees.

### 3.4. Studied Compounds

**Figure 4:**
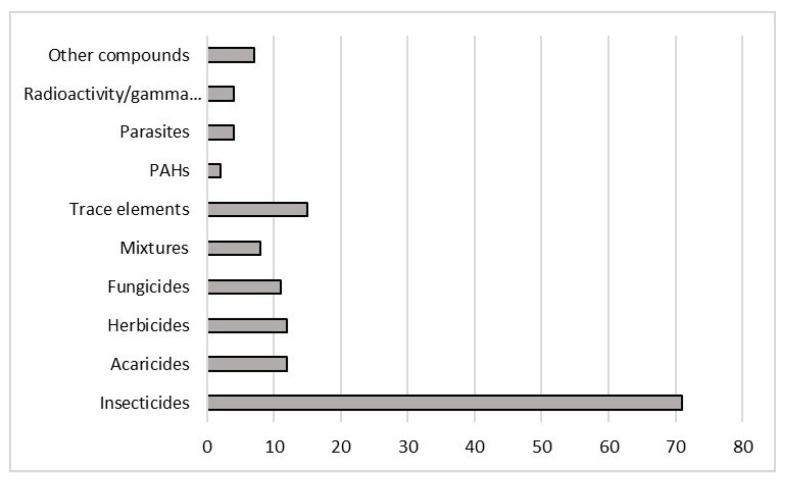
Number of studies, divided by kind of compounds, on *Apis mellifera*, that met the criteria for inclusion in this review.

Insecticides were investigated in 71 studies, followed by trace elements, in 15 papers. Studies on acaricides (12), herbicides (12), and fungicides (11) were present with a similar number. Mixtures and PAHs are still poorly studied, respectively with 8 and 2 papers (Figure 4). In the “other compounds” category, SO_2_, ethyl methane-sulfonate (EMS), ethanol and pharmaceutical compounds were included. In the category “parasites” are present not only papers that examined reactions to parasites but also other contaminants; there are not any papers that studied only parasites since they did not satisfy the criteria used for this review.

### 3.5. Effect Type

**Figure 5:**
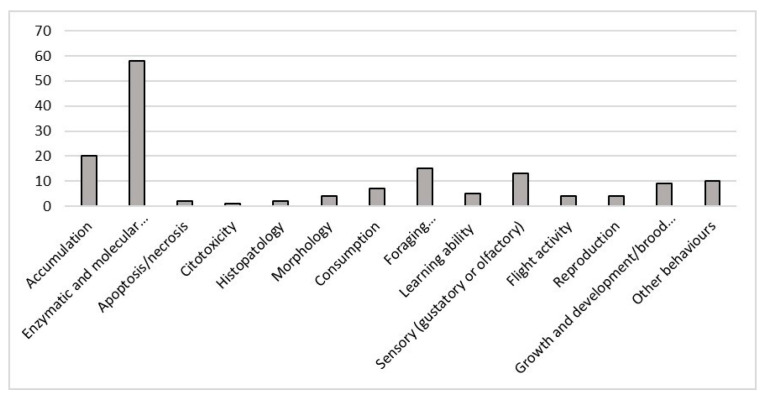
Number of studies, divided by kind of responses, on *Apis mellifera*, that met the criteria for inclusion in this review.

**Figure 6:**
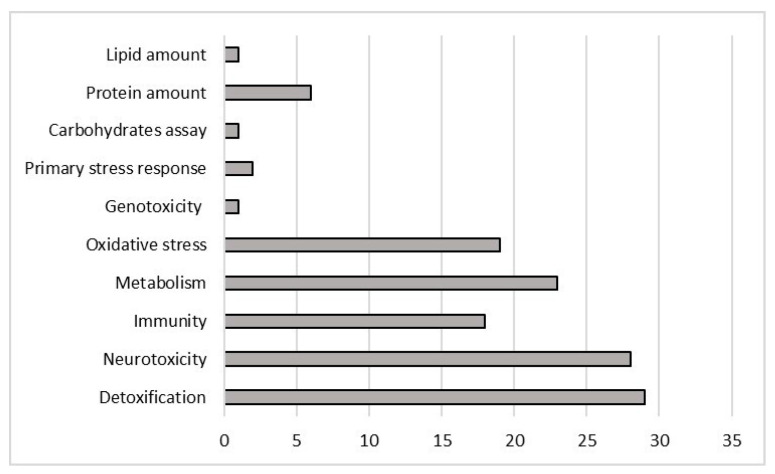
Number of studies, divided by molecular and enzymatic responses, on *Apis mellifera*, that met the criteria for inclusion in this review.

Most studies used for this review investigated more than one effect (64 studies) on honey bees but 42 studies concentrated on investigating just one effect. The most widely studied single effect type was accumulation (20) followed by foraging activity (15) studies (Figure 5). Figure 6 shows studies regarding enzymatic and molecular responses (58): the effects that were studied in more depth were detoxification (27) and neurotoxicity (26), followed by metabolic responses (21), immunity (17), and oxidative stress (15).

In the following tables, all the examined papers are summarized by endpoint; there are two tables for each methodological approach, one for cellular to whole organism and population endpoints, and one for molecular and enzymatic endpoints.

Endpoints examined in laboratory studies are summarized in Table 1 and Table 2. Table 1 shows two endpoints were most used in laboratory studies, “foraging activity/fitness/production of matrixes” and “sensory (gustatory or olfactory)”, both with a total of 12 papers.

Table 2 shows the molecular and enzymatic endpoints examined in laboratory studies. The most studied effect concerned “neurotoxicity” (24 studies) and the test that was applied most frequently was the acetylcholinesterase (AChE) activity; only two papers examined the presence of trembling, hyperactivity, and paralysis in the organisms exposed mostly to insecticides. The second most investigated endpoint was “detoxification”, with studies mostly concerning the activity of glutathione-S-transferase (GST) or CYP450. Another endpoint with a considerable number of papers (17) was “metabolism”, in which alkaline phosphatase (ALP) and ATPase were mostly examined. “Oxidative stress” endpoint was examined only in 14 papers, evaluating the activity of antioxidant enzymes such as catalase (CAT) and superoxide dismutase (SOD).

In semi-field studies, the most frequently studied endpoints are “foraging activity/fitness/production of matrixes” and “other behaviors”, both with 6 studies (Table 3). In Table 4 the molecular endpoints are summarized; in this case the most examined endpoints (3 studies) were “protein amount” and “immunity”, followed by “detoxification”, with 2 papers.

The endpoints examined in field studies are summarized in Table 5 and Table 6. Table 5 shows that 16 studies observed “accumulation” in the honey bees sampled in sites with different levels of anthropogenic pressure. In general, herbicides and insecticides were the contaminants that tended to be observed more in these accumulation studies.

Table 6 shows molecular endpoints examined in field studies. The effect that was studied with the highest degree of frequency concerned “detoxification” and “metabolism”, both with 5 papers. The next two endpoints that were examined with a good degree of frequency were “neurotoxicity” and “oxidative stress”; the first was observed through the evaluation of AChE activity, the second mostly with the observation of CAT and SOD activity.

## 4. Discussion

The exposure of honey bees to environmental pollutants, especially agrochemical products, is causing a decline in their colonies [11,144], leading also to consequences for crop production, food security, and environmental health. For this reason, it is important to understand primarily both the benefits and the risks that the use of PPPs pose to the environment in order to make decisions about agricultural management. To determine the role of pesticides and other contaminants and their impact on honey bees it is essential to understand the kind of studies that have been conducted until now.

The majority of studies into the effects of pollutants on bees have been undertaken in North America and Europe, where important honey bee colony losses have been reported [14,15,16]. However, this phenomenon should be studied globally, in order to ascertain a better understanding of its causes. Although, PPPs tend to be most widely used in developed countries, they are increasingly being used in other parts of the world where regulations and best practices around their environmental impacts may not be as stringent [145].

The great majority of examined papers were about adult honey bees; it would be useful for there to be an improvement in the studies conducted related to other life stages, in order to have a better understanding of whether and how environmental contaminants may affect every stage of a honey bee’s life cycle.

This review underlined that the majority of studies on honey bees are carried out in a laboratory more than in semi-field and field conditions, in a controlled environment and with controlled environmental exposure to the selected substances. The vast majority of papers about laboratory experiments reviewed focused on the sublethal effects, mostly about foraging activity, sensorial ability, neurotoxicity, detoxification, metabolism, and oxidative stress. In semi-field studies different responses both at macroscopic and microscopic levels were considered; however, in this review, only 14 papers of this kind were found. Honey bees, in the field, are exposed to multiple stressors and most of the field papers were monitoring studies where accumulation of various contaminants in *Apis mellifera* were investigated; only 8 papers [28,33,50,57,62,71,83,95] analysed the sublethal effects of the contaminant mixtures on *Apis mellifera*. All these studies highlighted that honey bees are sensitive bioindicators of environmental pollution. Therefore, it is only through context monitoring that the honey bees decline should be examined, in order to understand its causes and to provide effective prevention tools to administrations.

In this review, it is highlighted that the most widely investigated PPPs are insecticides, because they were demonstrated to be harmful to non-target organisms, such as honey bees. Different authors observed that neonicotinoid insecticides, such as imidacloprid, thiamethoxam, acetamiprid, dinotefuran, thiacloprid, nitenpyram, and clothianidin, are able to damage honey bees olfactory learning performances [65,76,78], foraging activity [65,68,69], and homing flight abilities [119]. This kind of compounds may cause neurotoxicity in honey bees, by altering AChEactivity which may be induced [97] or inhibited [84], and by modulating carboxylesterase (CaE) activity [53,82]. Furthermore, detoxification and antioxidant enzymes activities seem to be altered by neonicotinoids, such GST [53,81,82], CAT [53], PPO [84], ALP [53] and CYP450 [94] activities. Moreover, these compounds may affect the immune system for instance, by modulating the content of vitellogenin [47,101], by reducing the hemocytes density, encapsulation response and antimicrobial activity [83], and by modulating the relative abundance of several key gut microbial molecules [66]. Several authors studied the effects of pyrethroid insecticides, such as deltamethrin, bifenthrin, cypermethrin, permethrin, and λ-cyhalotrin, on honey bees; these compounds seems to cause neurotoxicity by increasing AChE activity [59,91], modulating CaE activity [55]. Pyrethroids caused variations in lipid [107] and carbohydrates [96], reduced learning, memory performances [62,72] and foraging activity [67], and influenced bees locomotion and social interaction [117]. This class of insecticides is also able to cause variations in metabolic and detoxification activities, such as increasing GST activity [89,136], modulating ALP activity [107], inducing the expression of CYP450 monooxygenase [86], and inhibiting Na^+^, K^+^-ATPase activity [96]. Moreover, they may induce immune responses, cause changes in the activity of POD and in the content of MDA and LPO and induce oxidative stress [59]. Authors, who studied organophosphorus insecticides effects, observed an inhibition in the odour learning [79], a modulation of AChE activity [75,77,84,95], a modulation of different immune system related genes and an induction of vitellogenin transcript [86]. El-Saad et al. (2017) [56] observed midguts ultrastructural modifications, a reduction of GSH levels, an inhibition of SOD, CAT and GPx activities, and an increase in MDA levels.

A recent review [146] underlined that other PPPs, such as fungicides and herbicides, that are not designed to target insects, may be factors that influence honey bees decline. For this reason, it would be important to increase the number of studies conducted related to their effects on these pollinators. Papers included in this review showed that the most frequently studied herbicide was glyphosate; it seems to cause a more indirect homing flight [122], to reduce sensitivity to sucrose and learning performance [69], to delay worker brood development [120], to have effects on the expression of CYP isoforms genes [87], and to slightly inhibit AChE activity [97].

Moreover, we believe that studies regarding other pollutants, such as PAHs and trace elements, should be improved, because of their presence in the environment that could cause honey bees exposure and adverse effects. Studies on trace elements underlined that pollutants, like aluminum, cadmium, selenium, lead, and copper, are able to influence foraging behavior [63,70] and the development time [51,66], to cause histopathological alterations [57], to alter AChE, ALP, GST [43,54], CAT and SOD [107,140] activities. The European Food Safety Authority (EFSA) pointed out that the study of the impact of mixtures of chemicals also compared to non-chemical stressors, like *Varroa destructor* and viruses, on honey bee health are of great relevance, in view to support the implementation of a holistic risk assessment method [147,148].

In field studies, it is more difficult to understand the effects caused by single contaminants, due to the presence of multiple stressors. Up to now, few papers have investigated the sublethal effects on honey bees in their natural conditions and habitats. Badiou-Bénéteau et al. (2013) [54] and Nikolić et al. (2015) [135] highlighted the presence of sublethal effects, characterized by oxidative stress and the induction of detoxification processes, in honey bees from more anthropized areas, due to the presence of neurotoxic pollutants, such as metals. Lupi et al. (2020) [44] observed that pesticide mixtures, characterized by the combination of fungicides, insecticides, and plant regulators, could cause an increase in Reactive Oxygen Species (ROS) that can inhibit AChE and CAT activities. An inhibition of some antioxidant stress biomarkers (GSH, SOD, CAT, GST) was also observed in specimens collected from anthropized areas [56]. Nicewicz et al. (2020) [141] observed the importance of defensin and HSP70 levels as indicators of urban multistress both at individual and colony levels. Further studies are needed to investigate the ecotoxicological status of honey bee colonies.

Another aspect to be pointed out is that in all three types of experimental conditions (laboratory, semi-filed and field), research studies have focused their attention on the development of some biomarkers to assess exposure to and the effects of contaminants on honey bees, such as esterases activity to evaluate neurotoxic effects, antioxidant enzymes activity, and predominantly CAT and SOD, together with detoxification reactions and metabolic activity. However, several responses, such as genotoxicity and immune system alteration, remain poorly explored and require an increased interest and a significant degree of effort to ensure that research studies are conducted. Colin et al. (2004) [116] observed, for example, that the suppression of the immune system may lead to a decrease in the individual performance and consequently in the population dynamics and the degree of disorders present in the colony. Moreover, Lazarov and Zhelyazkova (2019) [149] observed that *Varroa destructor* infestations are responsible for the weakening of honey bees’ immune system, which may lead to a pronounced susceptibility of honey bees to contaminant exposure. To the best of our knowledge, Caliani et al. (2021) [43] is the only study that has been conducted into genotoxicity and that has examined *Apis mellifera*; in this study, it was observed that there are not only compounds such as EMS, with known genotoxic effects; indeed, there are also Cd and fungicides that have effects on the presence of hemocytes nuclear abnormalities.

While we have investigated the range of research approaches that have been used to study potential effects of contaminants on honey bees and provided a summary of main investigated effects (Table 1, Table 2, Table 3, Table 4, Table 5 and Table 6), a full evaluation of effects direction was beyond the scope of this research. As there are 106 papers included in this review it is clear that there is an increasing corpus of literature that examines the effects of a wide range of compounds on bees. Only when certain research gaps are addressed, may this area benefit from a meta-analysis in the future to establish a clearer picture of the magnitude and direction of each effect.

## 5. Conclusions

The current review highlighted that *Apis mellifera* biological responses to external stressors were studied mostly in Europe and North America; consequently, there is a notable need to increase monitoring in other regions. Insecticides are widely studied compounds compared to other PPPs, or other classes such as e PAHs and trace elements. Laboratory studies are useful in order to determine the effects of specific compounds; however, field studies should be implemented, in order to gain a better understanding of the ecotoxicological status of *A. mellifera* in relation to environmental contamination patterns. Through the observation of the different responses examined by the authors, several gaps have been identified that should be addressed, particularly within enzymatic and molecular responses, such as those regarding immune system and genotoxicity. The development of an integrated approach, supported by statistical models could be vital, in order to combine responses at different levels, from molecular ones to the organism and the population. This could be a valid tool to evaluate the impact of contamination on these organisms and to support monitoring strategies not only at a scientific level, but also at a regulatory one.

## Figures and Tables

**Table 1:** Summary of laboratory studies divided by endpoint and contaminants.

Endpoint	Test	N	Contaminants	Reference
Morphology	Cellular structure of midgut cells	2	CdO and PbO nanoparticles, mixtures	Dabour et al., 2019 [57]
	Morphologies of antenna and hypopharyngeal glands		Herbicides, fungicides, insecticides, acaricides	Tomè et al., 2020 [58]
Apoptosis/necrosis	Apoptosis/necrosis	2	Trace elements, mixtures	Dabour et al., 2019 [57]
	Apoptosis		Insecticides	Qi et al., 2020 [59]
Histopathology	Midgut, hypopharyngeal and brain	2	Insecticides	de Castro et al., 2020 [60]
	Midgut		Insecticides	Oliveira et al., 2019 [61]
Cytotoxicity	Midgut, hypopharyngeal and brain	1	Insecticides	de Castro et al., 2020 [60]
Consumption	Food consumption	7	CdO and PbO nanoparticles, mixtures	Al Naggar et al., 2020 [62]
	Food consumption		Insecticides, fungicides, Acaricides	Decourtye et al., 2005 [63]
	Food consumption		Herbicides	Helmer et al., 2015 [64]
	Food consumption		Sodium selenate, seleno-DL-methionine, DL-methionine	Hladun et al., 2012 [65]
	Food consumption		Insecticides	Tong et al., 2019 [27]
	Food consumption		Insecticides, mixtures	Williamson and Wright 2013 [28]
	Food consumption		Insecticides	Zhu et al., 2020 [66]
Foraging activity/ fitness/ production of matrixes	Foraging activity	12	Insecticides	Decourtye et al., 2004 [67]
	Sucrose response threshold		Sodium selenate, seleno-DL-methionine, DL-methionine	Hladun et al., 2012 [65]
	Foraging activity		Sodium selenate, sodium selenite, seleno-L-cystine	Hladun et al., 2013 [51]
	Fitness and production of wax and honey		Metals, selenium	Hladun et al., 2016 [68]
	Foraging activity		Herbicides	Herbert et al., 2014 [69]
	Foraging activity		Radiation (cell phone)	Mixson et al., 2009 [52]
	Foraging behaviour		Insecticides	Morfin et al., 2019 [70]
	Foraging activity		Mixtures	Prado et al., 2019 [50]
	Foraging activity		Insecticides, *Bacillus thurigiensis*, mixtures	Renzi et al., 2016 [33]
	Foraging activity		Fungicides, insecticides, mixtures	Schmuck et al., 2003 [71]
	Foraging activity		Trace elements	Søvik et al., 2015 [72]
	Weight, duration of immature development		Herbicides, fungicides, insecticides, acaricides	Tomè et al., 2020 [58]
Learning ability	Olfactory learning		Insecticides, fungicides, acaricides	Decourtye et al., 2005 [63]
	Visual and olfactory learning	4	Insecticides	Guez et al., 2010 [73]
	Training for olfactory conditioning using proboscis extension reflex		Insecticides, mixtures	Williamson and Wright 2013 [28]
	Learning and memory-related genes		Insecticides	Zhang et al., 2020 [74]
Other behaviours	Colony strength	5	Trace elements, selenium	Hladun et al., 2016 [68]
	Aggressive behaviour		Radiation (cell phone)	Mixson et al., 2009 [52]
	Hygienic behaviour		Insecticides	Morfin et al., 2019 [70]
	Thermoregulation		Insecticides	Tong et al., 2019 [27]
	Behavioural anomalies (exaggerated motility, discoordinated movements)		Fungicides, insecticides, mixtures	Schmuck et al., 2003 [71]
Reproduction	Viability of sperm		Insecticides, acaricides	Chaimanee et al., 2016 [75]
	Fecundity	3	Insecticides	Dai et al., 2010 [76]
	Prepupal weight, percentage of prepupation, and pupation, relative growth indices		Sodium selenate, sodium selenite, seleno-L-cystine	Hladun et al., 2013 [51]
Sensory (gustatory or olfactory)	Olfactory conditioning of Proboscis extension reflex (PER)	12	Insecticides	Al Naggar et al., 2015 [77]
	PER		Insecticides, acaricides	Decourtye et al., 2004 [67]
	PER		Insecticides, fungicides, acaricides	Decourtye et al., 2005 [63]
	PER		Insecticides	Guez et al., 2010 [73]
	Antennal response assays, Proboscis response assays		Sodium selenate, seleno-DL-methionine, DL-methionine	Hladun et al., 2012 [65]
	PER		Herbicides	Herbert et al., 2014 [69]
	PER		Insecticides	Imran et al., 2019 [78]
	PER		Radiation (cell phone)	Mixson et al., 2009 [52]
	PER		Insecticides, acaricides	Weick and Thorn 2002 [79]
	PER		Insecticides, mixtures	Williamson and Wright 2013 [28]
	PER		Insecticides	Wright et al., 2015 [80]
	PER		Insecticides	Yang et al., 2012 [81]
Flight activity	Flight navigation	3	Radiation (cell phone)	Mixson et al., 2009 [52]
	Flight ability and success		Insecticides	Tong et al., 2019 [27]
	Flight activity		Mixtures	Prado et al., 2019 [50]
Growth and development/brood production	Growth of adult workers	5	Insecticides, *Varroa destructor*	Abbo et al., 2017 [47]
	Growth and development		Insecticides	Dai et al., 2010 [76]
	Larval growth and development		Insecticides	du Rand et al., 2017 [82]
	Brood production		Trace elements, selenium	Hladun et al., 2016 [68]
	Duration of immature development		Herbicides, fungicides, insecticides, acaricides	Tomè et al., 2020 [58]
Accumulation	Chemical analysis	2	Sodium selenate, sodium selenite, seleno-L-cystine	Hladun et al., 2013 [51]
	Chemical analysis		Trace elements, selenium	Hladun et al., 2016 [68]

**Table 2:** Summary of laboratory studies divided by molecular and enzymatic endpoint and contaminants.

Endpoint	Test	n	Contaminants	Reference
Detoxification	CYP genes expression, glutathione-S-transferase (GST) genes expression	23	Insecticides	Al Naggar et al., 2015 [77]
	CYP and GST genes expression		CdO and PbO nanoparticles, mixtures	Al Naggar et al., 2020 [62]
	(GST)		Insecticides, fungicides, herbicides and mixture	Almasri et al., 2020 [83]
	GST		Insecticides	Badawy et al., 2015 [84]
	GST and CaEs		Insecticides	Badiou-Bénéteau et al., 2012 [53]
	GST and CaE		Fungicides, metals, EMS	Caliani et al., 2021 [43]
	GST		Insecticides	Carvalho et al., 2013 [55]
	Detoxification genes expression		Insecticides, acaricides	Chaimanee et al., 2016 [75]
	Genes encoding CYP450 monooxygenases		Insecticides	Christen et al., 2019 [85]
	Genes encoding CYP450 monooxygenases		Insecticides	Christen et al., 2019 [86]
	Proteomic and metabolomic analysis		Insecticides	du Rand et al., 2017 [82]
	Detoxification genes expression		Herbicides, fungicides, insecticides, *Varroa destructor*	Gregorc et al., 2012 [87]
	cytochrome P450 (CYP450), GST and CaEs		Insecticides, acaricides	Johnson et al., 2006 [88]
	CYP450		Insecticides, acaricides	Johnson et al., 2009 [89]
	GST and CaE		Insecticides	Li et al., 2017 [90]
	P450 genes expression		Acaricides	Mao et al., 2011 [91]
	GST isoenzymes expression			Papadopoulos et al., 2004 [92]
	GST, GR and gene expressions		Insecticides	Qi et al., 2020 [59]
	GST		Insecticides, *Bacillus thurigiensis*, mixtures	Renzi et al., 2016 [33]
	P450 genes expression		Herbicides, fungicides, insecticides, acaricides	Tomè et al., 2020 [58]
	Esterase (EST), GST, CYP450. CYPs and GSTs transcript levels		Insecticide	Yao et al., 2018 [93]
	CYP450 and phospholipase A2		Insecticides	Zaworra and Nauen 2019 [94]
	Detoxification genes expression		Insecticides	Zhu et al., 2020 [66]
Neurotoxicity	acetylcholinesterase (AChE)	24	Insecticides	Al Naggar et al., 2015 [77]
	AChE			Al Naggar et al., 2020
	AChE and CaE-3		Insecticides, fungicides, herbicides and mixture	Almasri et al., 2020 [83]
	AChE		Insecticides	Badawy et al., 2015 [84]
	AChE		Acaricides, mixtures	Badiou et al., 2008 [95]
	AChE and CaEs		Insecticides	Badiou-Bénéteau et al., 2012 [53]
	AChE		Insecticides	Bendahou et al., 1999 [96]
	AChE		Herbicides, insecticides	Boily et al., 2013 [97]
	AChE and CaE		Fungicides, trace elements, EMS	Caliani et al., 2021 [43]
	AChE and CaEs		Insecticides	Carvalho et al., 2013 [55]
	Genes encoding acetylcholine receptors		Insecticides	Christen et al., 2019 [85]
	Genes encoding acetylcholine receptors		Insecticides	Christen et al., 2019 [86]
	Trembling and paralysis		Insecticides, acaricides	Decourtye et al., 2004 [67]
	AChE and CaEs		Gamma irradiation	Gagnaire et al., 2019 [98]
	AChE		Insecticides	Glavan et al., 2018 [99]
	Esterase		Insecticides	Hashimoto et al., 2003 [100]
	AChE and CaE		Insecticides	Li et al., 2017 [90]
	AChE		Insecticides	Qi et al., 2020 [59]
	AChE		Insecticides	Rabea et al., 2010 [49]
	Octopamine, serotonin, dopamine		Trace elements	Søvik et al., 2015 [72]
	Hyperresponsiveness, hyperactivity and trembling		Insecticides	Suchail et al., 2001 [101]
	Protein level of synapsin		Insecticides	Tavares et al., 2019 [102]
	AChE		Insecticides, acaricides	Weick and Thorn 2002 [79]
	AChE		Insecticide	Yao et al., 2018 [93]
Immunity	Vtg expression	13	Insecticides, *Varroa destructor*	Abbo et al., 2017 [47]
	Defensin 1, Abaecin, Hymenoptaecin expressions		Insecticides	Al Naggar et al., 2015 [77]
	Nodulation		Dexamethasone (eicosanoid biosynthesis inhibitor)	Bedick et al., 2001 [103]
	Hemocytes density, encapsulation response and antimicrobic activity		Insecticides	Brandt et al., 2016 [104]
	Lysozyme (LYS) and granulocytes count		Fungicides, metals, EMS	Caliani et al., 2021 [43]
	Immune response genes expression		Insecticides, acaricides	Chaimanee et al., 2016 [75]
	Vtg gene expression		Insecticides	Christen et al., 2019 [105]
	Vtg gene expression		Insecticides	Christen et al., 2019 [86]
	Phenoloxydase (PO)		Gamma irradiation	Gagnaire et al., 2019 [98]
	Immune genes expression		Herbicides, fungicides, insecticides, *Varroa destructor*	Gregorc et al., 2012 [87]
	Immune gene expression		Insecticides	Li et al., 2017 [90]
	Vtg synthesis		Insecticides	Pinto et al., 2000 [106]
	Immune genes expression		Insecticides	Zhu et al., 2020 [66]
Metabolism	Alkaline phosphatase (ALP) and GST		Insecticides, fungicides, herbicides and mixture	Almasri et al., 2020 [83]
	Alkaline phosphatase (ALP) and GST	17	Insecticides	Badiou-Bénéteau et al., 2012 [53]
	Na^+^, K^+^ -ATPase assay		Insecticides	Bendahou et al., 1999 [96]
	ALP		Insecticides	Bounias, 1985 [107]
	ALP and GST		Fungicides, metals, EMS	Caliani et al., 2021 [43]
	ALP and GST		Insecticides	Carvalho et al., 2013 [55]
	Genes encoding for enzymes involved in phosphorylation		Insecticides	Christen et al., 2019 [85]
	Proteomic and metabolomic analysis		Insecticides	du Rand et al., 2017 [82]
	GST, CaEs and ALP		Gamma irradiation	Gagnaire et al., 2019 [98]
	GST and CaE		Insecticides	Li et al., 2017 [90]
	Aspartate aminotransferase (AST), alanine aminotransferase (ALT), ALP		Insecticides	Paleolog et al., 2020 [108]
	ATP assays and GADPH activity		Mixtures	Prado et al., 2019 [50]
	ATPase		Insecticides	Rabea et al., 2010 [49]
	GST, ALP		Insecticides, *Bacillus thurigiensis*, mixtures	Renzi et al., 2016 [33]
	Metabolic profile		Insecticides	Shi et al., 2018 [109]
	AST, ALT, ALP		Acaricides	Strachecka et al., 2016 [110]
	Abundance of gut microbiota for metabolic homeostasis, metabolic genes expression		Insecticides	Zhu et al., 2020 [66]
Oxidative stress	GST, G6PDH		Insecticides, fungicides, herbicides and mixture	Almasri et al., 2020 [83]
	GST, superoxide dismutase (SOD) and catalase (CAT) genes expression		CdO and PbO nanoparticles, mixtures	Al Naggar et al., 2020 [62]
	polyphenol oxidase (PPO)	14	Insecticides	Badawy et al., 2015 [84]
	CAT		Insecticides	Badiou-Bénéteau et al., 2012 [53]
	CAT		Insecticides	Carvalho et al., 2013 [55]
	CAT, SOD, glutathione peroxidase (GPx), GST		Gamma irradiation	Gagnaire et al., 2019 [98]
	α-tocopherol and metallothionein-like proteins (MTLPs)		Trace elements	Gauthier et al., 2016 [111]
	LPO, lutein, zeaxanthin, α-Cryptoxanthin, β-Cryptoxanthin, β-Carotene, at-ROH, α-Tocopherol		Herbicides	Helmer et al., 2015 [64]
	GST and PPO		Insecticides	Li et al., 2017 [90]
	SOD, CAT, reduced glutathione (GSH), protein thiol groups (SH), malondialdehyde (MDA)		Trace elements	Nikolić et al., 2016 [112]
	DNA methylation		Insecticides	Paleolog et al., 2020 [108]
	Peroxidase (POD), malondialdehyde (MDA), lipid peroxide (LPO), SOD, CAT		Insecticides	Qi et al., 2020 [59]
	GAPD, G6PD		Insecticides, *Bacillus thurigiensis*, mixtures	Renzi et al., 2016 [33]
	SOD, GPx, CAT, GST		Acaricides	Strachecka et al., 2016 [110]
Genotoxicity	Nuclear abnormalities (NA) assay	1	Fungicides, metals, EMS	Caliani et al., 2021 [43]
Primary stress response	HSP70	1	Ethanol	Hranitz et al., 2010 [113]
Carbohydrates assay		2	Insecticides	Bendahou et al., 1999 [96]
			Insecticides	Bounias, 1985 [107]
Protein amount		3	Herbicides	Helmer et al., 2015 [64]
			Insecticides	Li et al., 2017 [90]
			Insecticides	Pinto et al., 2000 [106]
Lipid amount		1		Bounias, 1985 [107]

**Table 3:** Summary of semi-field studies divided by endpoint and contaminants.

Endpoint	Test	n	Contaminants	Reference
Morphology	Asymmetry of wing nervature, diameter of forager bee hypopharyngeal gland, asymmetry of left and right branches of ovary	1	Insecticides	Wegener et al., 2016 [114]
Foraging activity/ fitness/production of matrixes	Colony nutritional status	6	Acaricides	Cabbri et al., 2018 [115]
	Foraging activity		Insecticides	Colin et al., 2004 [116]
	Foraging activity		Insecticides, acaricides	Decourtye et al., 2004 [67]
	Time spent near a food source		Insecticides	Ingram et al., 2015 [117]
	Foraging activity		Fungicides, insecticides	Schmuck et al., 2003 [71]
	Foraging behaviour		Insecticides	Shi et al., 2020 [118]
Learning ability	Learning capacity and long-term memory of presumed forager bees	1	Insecticides	Wegener et al., 2016 [114]
Other behaviours	Intensive cleaning, trembling, cramping, locomotion problems, inactive bees, aggressiveness	6	Fungicides, insecticides	Berg et al., 2018 [48]
	Bee locomotion and social interactions		Insecticides	Ingram et al., 2015 [117]
	Homing performances		Insecticides	Monchanin et al., 2019 [119]
	Overwintering success		Herbicides	Odemer et al., 2020 [120]
	Overwintering success		Insecticides	Siede et al., 2017 [121]
	Behavioural anomalies (exaggerated motility, discoordinated movements, trembling, shaking, apathy)		Fungicides, insecticides	Schmuck et al., 2003 [71]
Reproduction	Number of capped brood cells	1	Insecticides	Wegener et al., 2016 [114]
Sensory (gustatory or olfactory)	PER	1	Insecticides, acaricides	Decourtye et al., 2004 [67]
Flight activity	Homeward flight path	2	Herbicides	Balbuena et al., 2015 [122]
	Flight activity		Fungicides, insecticides	Berg et al., 2018 [48]
Growth and development/brood production	Development of bee brood	4	Fungicides, insecticides	Berg et al., 2018 [48]
	Brood and colony development, colony weight		Herbicides	Odemer et al., 2020 [120]
	Number of brood cells, weight gain and production of drones		Insecticides	Siede et al., 2017 [121]
	Reduction in bees and brood		Insecticides	Thompson et al., 2019 [123]
Accumulation	Chemical analysis	1	Insecticides	Siede et al., 2017 [121]

**Table 4:** Summary of semi-field studies divided by molecular and enzymatic endpoint and contaminants.

Endpoint	Test	n	Contaminants	Reference
Detoxification	GST	2	Insecticides	Wegener et al., 2016 [114]
	CYP450, CaEs, GST		Insecticides	Zhu et al., 2020 [124]
Neurotoxicity	Trembling and paralysis	1	Insecticides	Decourtye et al., 2004 [67]
Immunity	Vtg and apolipophorin (APO)	3	Acaricides	Cabbri et al., 2018 [115]
	Hymenoptaecin gene expression		Insecticide	Siede et al., 2017 [121]
	Vtg		Insecticides	Wegener et al., 2016 [114]
Metabolism	Phosphofructokinase	1	Insecticides	Wegener et al., 2016 [114]
Oxidative stress	GST, phenoloxydase, glucose oxidase	1	Insecticides	Wegener et al., 2016 [114]
Protein amount		3	Acaricides	Cabbri et al., 2018 [115]
			Insecticides	Wegener et al., 2016 [114]
			Insecticides	Zhu et al., 2020 [124]

**Table 5:** Summary of field studies divided by endpoint and contaminants.

Endpoint	Test	n	Contaminants	Reference
Morphology	Wing asymmetry	1	Urbanisation	Leonard et al., 2018 [125]
Accumulation	Chemical analysis	18	Metals	Al Naggar et al., 2013 [126]
	Chemical analysis		Insecticides	Al Naggar et al., 2015 [127]
	Chemical analysis		Insecticides	Al Naggar et al., 2015 [128]
	Chemical analysis		PAHs	Amorena et al., 2009 [129]
	Chemical analysis		Fungicides, insecticides	Amulen et al., 2017 [130]
	Chemical analysis		Insecticides	Codling et al., 2016 [131]
	Chemical analysis		Metals	Conti and Botrè, 2001 [132]
	Chemical analysis		Insecticides	El-Saad et al., 2017 [56]
	Chemical analysis		Herbicides, insecticides	Fulton et al., 2019 [133]
	Chemical analysis		Metals	Kump et al., 1996 [134]
	Chemical analysis		Herbicides, fungicides, insecticides, acaricides	Mullin et al., 2010 [45]
	Chemical analysis		Trace elements	Nikolić et al., 2015 [135]
	Chemical analysis		PAHs	Perugini et al., 2009 [136]
	Chemical analysis		SO_2_	Ponikvar et al., 2005 [137]
	Chemical analysis		Herbicides, fungicides, insecticides	Raimets et al., 2020 [46]
	Chemical analysis		Herbicides, insecticides, metals	Ruschioni et al., 2013 [138]
	Gamma spectrometry		Radiations	Tonelli et al., 1990 [139]
	Chemical analysis		Trace elements	van der Steen et al., 2012 [140]

**Table 6:** Summary of field studies divided by molecular and enzymatic endpoint and contaminants.

Endpoint	Test	n	Contaminants	Reference
Detoxification	GST and metallothioneins (MT)	4	Trace elements	Badiou-Bénéteau et al., 2013 [54]
	GST		Herbicides, fungicides, insecticides, electromagnetic fields	Lupi et al., 2020 [44]
	GST		suspended dust and heavy metals	Nicewicz et al., 2020 [141]
	GST, esterases, epoxyde hydrolase and DDT-dehydrochlorinase		Insecticides	Yu et al., 1984 [142]
Neurotoxicity	AChE	4	Trace elements	Badiou-Bénéteau et al., 2013 [54]
	AChE		Herbicides, fungicides, insecticides, electromagnetic fields	Lupi et al., 2020 [44]
	AChE		suspended dust and heavy metals	Nicewicz et al., 2020 [141]
	Esterases		Insecticides	Yu et al., 1984 [142]
Immunity	Defensin	1	suspended dust and heavy metals	Nicewicz et al., 2020 [141]
Metabolism	ALP and GST	5	Trace elements	Badiou-Bénéteau et al., 2013 [54]
	ALP and Acidic phosphatase		Trace elements	Bounias et al., 1996 [143]
	ALP and GST		Herbicides, fungicides, insecticides, electromagnetic fields	Lupi et al., 2020 [44]
	GST		suspended dust and heavy metals	Nicewicz et al., 2020 [141]
	GST			Yu et al., 1984 [142]
Oxidative stress	SOD, CAT, GPx, GR	4	Insecticides	El-Saad et al., 2017 [56]
	SOD and CAT		Trace elements	Nikolić et al., 2015 [135]
	CAT and GST		Herbicides, fungicides, insecticides, electromagnetic fields	Lupi et al., 2020 [44]
	GST and total antioxidant capacity (TAC)		suspended dust and heavy metals	Nicewicz et al., 2020 [141]
Primary stress response	HSP70	1	suspended dust and heavy metals	Nicewicz et al., 2020 [141]

## Data Availability

Data sharing not applicable.
